# Evaluation of the effects of green tea extract as a dietary supplement in sheep on gas production, volatile fatty acids, and digestibility

**DOI:** 10.14202/vetworld.2024.2204-2210

**Published:** 2024-10-04

**Authors:** Ririn Siti Rahmatillah, Diky Ramdani, Iman Hernaman, Anuraga Jayanegara, Nanik Hidayatik

**Affiliations:** 1 Department of Animal Production, Faculty of Animal Husbandry, Universitas Padjadjaran, Sumedang, West Java, 45363, Indonesia; 2 Department of Animal Nutrition and Feed Technology, Faculty of Animal Husbandry, Universitas Padjadjaran, Jatinangor Campus, Sumedang 45363, Indonesia; 3 Department of Animal Nutrition and Feed Technology, Faculty of Animal Husbandry, IPB University, Bogor, 16680 Indonesia; 4 Department of Veterinary Science, Faculty of Veterinary Medicine, Universitas Airlangga, Surabaya, 60132 Indonesia

**Keywords:** *Camellia sinensis*, Garut sheep, green tea extract, *in vitro*

## Abstract

**Background and Aim:**

Phenolic tannins, which are ubiquitous in plants, exhibit diverse biological activities and have drawn significant attention for their potential impact on ruminant nutrition and health. Although phenolic tannins have beneficial and detrimental effects on rumen fermentation, their precise influence remains poorly understood. This study aimed to explore the effects of varying doses of green tea extract (GTE) on rumen fermentation parameters through an *in vitro* trial using sheep rumen liquids.

**Materials and Methods:**

A 4 × 2 factorial design was used to test the effect of 4 different doses of GTE treatment (0, 140, 280, and 560 mg/kg) in 2 different *in vitro* runs on degradability, fermentation profiles, and gas production using the *in vitro* Hohenheim Gas Test method.

**Results:**

Across running times, the GTE-treated diet did not affect (p > 0.05) dry matter degradability % and organic matter degradability %, pH, ammonia (NH_3_-N, mg/dL), 24 h total gas production (tGP 24h, mL), and acetate-to-propionate ratio (A: P), but it reduced (p < 0.05) tGP 6 h compared with GTE-0 (control diet without GTE). GTE treatment tended to reduce (p < 0.1) methane (CH_4_, % LEL) and total volatile Fatty Acids (tVFA, mM). Across GTE treatment, the 1^st^ *in vitro* run had higher (p < 0.001) tGP 6–24 h and pH, but lower (p < 0.001) tVFA and A: P in comparison with the 2^nd^ *in vitro* run.

**Conclusion:**

GTE treatment tends to decrease CH_4_ output in rumen without affecting degradability, tGP, and most fermentation profiles, except for a tendency to reduce tVFA.

## Introduction

Sheep farming plays a crucial role in supporting the livelihoods of many rural communities. However, the challenges associated with the nutritional requirements of these animals persist, primarily due to the prevalent use of low-nutrient feeds such as paddy straw, which is both economical and readily available [1, 2].

To increase sheep productivity, one promising avenue is the use of natural dietary additives with potential health benefits for livestock. Green tea extract (GTE), renowned for its high polyphenol content, has emerged as a candidate worthy of exploration. Polyphenols, known for their antioxidant and anti-inflammatory properties, have demonstrated beneficial effects on various aspects of animal health and performance [2, 3]. The potential impact of GTE on *in vitro* rumen fermentation parameters is an area of interest, as it can offer insights into the enhancement of nutrient utilization and overall digestive efficiency in sheep. The effects of flavonoids, including catechins found predominantly in tea, have been studied in a wide range of biological activities along with their effects on the promotion of health and prevention of diseases in humans [4, 5]. These actions are almost certainly mediated in part by the radical scavenging and antioxidant activities of tea polyphenols [6].

This study aimed to explore the bioactive components of GTE and investigate the effects of adding GTE to sheep diets on critical rumen degradability and fermentation parameters, including total gas production (tGP) and methane (CH_4_) production, pH levels, individual volatile fatty acids (VFA), and ammonia (NH_3_-N) concentrations. By delving into the influence of GTE on these key parameters, we aimed to contribute valuable information that can be applied to optimize the nutritional management of sheep. This research addresses the nutritional challenges associated with prevailing feeding practices and offers a sustainable and accessible solution for improving the health and productivity of sheep in resource-constrained environments.

## Materials and Methods

### Ethical approval

The use of slaughtered local lambs as rumen fluid (RF) donors in this experiment was approved by the Padjadjaran University Research Ethics Commission (number: 1337/UN6.KEP/EC/2023).

### Study period and location

The maceration process for the original tea leaves was conducted at the Biotechnology Research and Testing Laboratory of the Faculty of Animal Husbandry Universitas Padjadjaran on October 27, 2023, followed by evaporation at the Central Laboratory of Universitas Padjadjaran on October 30, 2023. The *in vitro* incubation, pH, CH_4_, dry matter degradability, and organic matter degradability assessments were performed from December 25, 2023, to January 15, 2024, at the Biotechnology Research and Testing Laboratory of the Faculty of Animal Husbandry. The VFA measurement was conducted at the Central Laboratory of Universitas Padjadjaran on January 16, 2024. The NH_3_ analysis was carried out on January 23, 2024, at the National Research and Innovation Agency (BRIN) in Cibinong, Bogor.

### GTE and dietary preparations

Steamed green tea leaves were obtained from The Gambung Tea and Quinine Research Center, Bandung, West Java, Indonesia. The original tea leaves were then extracted using an ethanol–water solvent at a concentration ratio of 50% and a maceration time of 24 h with 2 rinses. After maceration, the GTE solution was evaporated using a rotary evaporator at a temperature of 45°C and 0.28 × *g* until a thick GTE layer was obtained and used for treatment.

A 4 × 2 factorial design was used to test the impacts of 4 different doses of GTE treatment (0, 140, 280, and 560 mg/kg) in 2 different *in vitro* running times on *in vitro* degradability, tGP, and fermentation profiles using 6 replicates divided into 2 *in vitro* runs. The basal diet was prepared by mixing commercial concentrate and paddy straw at a 60:40 ratio. The concentrate concentration consisted of 21% palm kernel meal, 19.5% dried cassava, 15% rice bran, 11% coffee skin, 9% copra meal, 8.5% molasses, 6% cocoa shell, 3.95% cassava, 2% soybean meal, 2% premix (Lagantor F1 Costumix, Kalbe Animal Health), 2% lime, 1.5% Rapeseed meal, 1% distillers dried grains, 1% corn germ feed, 0.5% wheat pollard, 0.3% sodium bicarbonate, and 0.2% salt.

### Buffered innoculum

RF was collected for each *in vitro* run from three local lambs from a slaughterhouse in Parongpong, Bandung, West Java. The lambs had a similar slaughter weight and feeding history as those from the same company. Immediately after slaughter, the rumen was cut, and the RF was immediately filtered through two layers of muslin cloth in a large funnel connected to warm insulated flasks (3 flasks for 3 RF of lambs) until filled and tightly closed, supporting anaerobic conditions for later storage. The samples were transported to the laboratory for immediate use within 1 h of collection. After that, each RF was appropriately measured and transferred quickly, under the filtration of two layers of muslin cloth, into a pre-warmed dark bottle (capacity 2.5 L) containing a buffer solution [7] in a ratio of 1:2 (RF: buffer) while stored in a water bath (39°C). The RF-buffered inoculum bottle was then flushed with CO_2_ to remove oxygen and closed tightly with a dispenser. The pH of each RF-buffered inoculum was adjusted to around 7 ± 0.2 using dropwise HCl.

### *In vitro* incubation

Approximately 300 ± 6 mg of each sample was transferred into a 100 mL glass syringe (Fortuna Optima Poulten Graf, Wertheim, Germany), thinly lubricated with saline, and equipped with a straight stopcock. Approximately 30 mL of the RF-buffered inoculum was added to each glass syringe and placed in a shaking water bath at 39°C. The incubation process was conducted in two runs, each using the same homogeneous mixture of RFs from three sheep, with each treatment consisting of six replicate syringes (n = 6). The tGP in each syringe was measured every 2 h for 24 h. After incubation, most of the warm water in the water bath was replaced with sufficient ice to stop further fermentation in the syringes. Approximately 20 mL of gas from each incubated syringe was then transferred to another clean syringe with a stopcock for direct CH_4_ analysis. Next, all contents in each syringe (inoculum and residue) were transferred into a previously weighed tube (polyethylene, 50 mL capacity) for measurement of pH and *in vitro* dry matter degradability (DMD). pH was measured directly using a pH meter. All tubes were then centrifuged, and samples were prepared for analysis of VFA and NH_3_-N. All residual particles remaining in the syringe were washed with water and placed into a suitable tube containing the residue. This undigested residue was dried at 60°C using a drying oven for DMD determination, as described by Khan and Chaudhry [8].

### Degradability and fermentation profile analyses

Organic matter (OM) was analyzed using the following procedure: each dry matter (DM) sample was placed and ignited in a furnace (Thermo Fisher Scientific Indonesia) with the temperature raised slowly to 550°C for 5 h. The sample was further removed and cooled in a desiccator before being weighed. The OM degradability (OMD, %) was obtained by estimating the difference between OM in the diet and OM in the residue and expressed as the percentage degradability. DMD (%) was calculated as a percentage of the weight loss of the feed sample. The samples were placed in an oven at 60°C for 48 h for DM analysis. The NH_3_ concentration was measured using the method of Sarwono [9] for NH_3_-N measurement in RF with the help of a spectrophotometer. The CH_4_ (%) in tGP was measured using a Riken Keiki (RKI, Japan) GX-2012 Confined Space Gas Monitor in the first 24 h of the gas produced by the *in vitro* process. After incubation, samples were preserved using H_3_PO_4_ for individual VFA analysis using a method similar to that used by Ramdani *et al*. [10]. Samples were analyzed using gas chromatography-mass spectrometry (GC/MS) (Agilent 7890A, USA), a detector (MS Agilent 5977B GC/MS), and a column (Agilent, 123-3262 DB-FFAP) with a length of 60 m, a diameter of 0.325 mm, and a film thickness of 0.25 um. The mobile phase was in the form of Helium Gas at a speed of 1 mL/min. The injector temperature is 160°C, and the column temperature of 80°C is held for 1 min, resulting in an increase of 15°C/min to a temperature of 115°C and held for 3 min; after that, it rises 3°C/min to a temperature of 130°C, then rises 15°C/min to a temperature of 230°C and holds for 3 min. The ratio of split injection is 15:1. Individual VFA values were calculated based on the value of the sample peak area compared to the standard single peak area (Volatile free acid mix; Supelco. CRM46975, USA) containing 10.00 mM acetic acid, propionic acid, isobutyric acid, butyric acid, isovaleric acid, and valeric acid.

### Chemical analysis

Each sample of paddy straw silage or concentrate was randomly sampled from three or five different blue plastic barrels or sacks and pooled. Each sample was dried in an oven at 60°C for approximately 48 h. Each dried sample was then ground and passed through a 1-mm sieve in a sample disc mill before being subjected to various nutrient analyses using standard protocols of the Association of Official Analytical Collaboration [11] to determine crude protein (CP, AOAC 990.03), ash (AOAC 942.05), and ether extract (EE, AOAC 920.39). The neutral detergent fiber (om) content was analyzed using the procedure described by Van Soest *et al*. [12] without the use of amylase and decalin, whereas acid detergent fiber (om) content was determined using the method of Van Soest [13]. The metabolic energy (ME) was analyzed using the method of Menke and Steingass, as described by Ramdani *et al*. [10]. Total phenols and total tannins were analyzed using the Folin-Ciocalteu method [14, 15]. All nutrient contents were expressed as percentage DM, except for DM and ME, which were expressed as percentages of fresh samples and MJ/kg, respectively (Table-1).

**Table-1:** Mean proximate contents (%dry matter or otherwise stated, n=2) of feed materials.

Nutrients	Concentrate (%)	Paddy straw silage (%)	Tea extract
Dry matter	88.7	35.9	-
Organic matter	88.8	88.9	-
Crude protein	14.7	7.33	-
Ether extract	4.88	3.04	-
ADF	28.0	38.2	-
NDF	56.2	44.4	-
Metabolizable energy	6.70 MJ/kg	3.48 MJ/kg	-
Total phenol	-	-	79.6%
Total tannins	1.75	4.80	47.1%

ADF=Acid detergent fiber, NDF=Neutral detergent fiber

### Statistical analysis

The data were statistically analyzed using the General Linear Model in Minitab 16 statistical software (https://www.minitab.com/en-us/products/minitab/), in which Tukey’s test was applied to compare means. Statistical significance was set at p ≤ 0.05. The residual data were analyzed for normality by passing the Anderson-Darling normality test at p > 0.05 [16].

## Results

**Figure-1:**
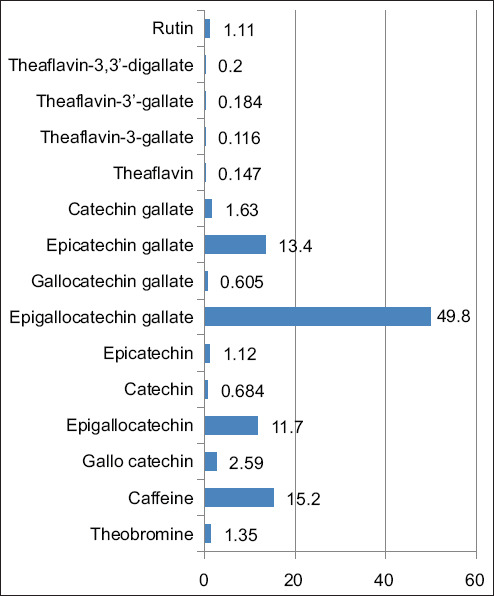
Plant bioactive components of green tea extract (*Camelia sinensis* var. *asamica*) are expressed as percentages of total identified bioactive compounds [15].

**Figure-2:**
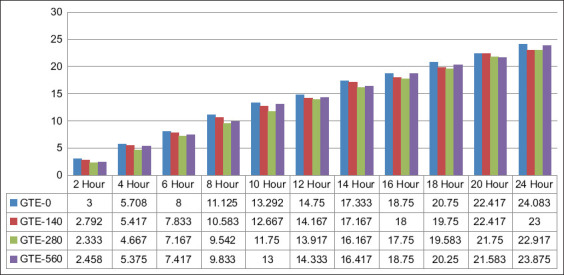
Gas production patterns of GTE-0, GTE-140, GTE-280, and GTE-560 (mL) during 24 h incubation. GTE=Green tea extract.

Figure-1 shows that the main components identified include caffeine content at 15.2%, epigallocatechin gallate (EGCG) at 49.8%, epigallocatechin at 11.7%, and epicatechin gallate at 13.4% [15]. Apart from that, there is also theobromine with a level of 1.35%, routine with a level of 1.11%, and several other catechins such as catechin, gallocatechin gallate, and catechin gallate in lower amounts. Other components include epicatechin and various flavin derivatives such as the aflavin-3-gallate, theaflavin-3’-gallate, and theaflavin-3,3’-digallate, all of which are present in fairly small amounts in GTE. The results of the *in vitro* trial (Table-2) show that varying doses of GTE added to a sheep diet did not have a significant effect (p > 0.05, Table-2) on tGP 12-24h, IVDMD, IVOMD, pH, CH_4_, NH_3_, and VFA during a 24-h *in vitro* incubation. Across running times, the GTE-treated diet did not affect (p > 0.05) dry (DMD, %) and organic (OMD, %) matters digestibility, pH, ammonia (NH_3_-N, mg/dL), 24 h tGP (tGP 24 h, mL), and acetate-to-propionate ratio (A: P), but it reduced (p < 0.05) tGP 6 h compared with GTE-0 (control diet without GTE). GTE treatment tended to reduce (p < 0.1) methane (CH_4_, % LEL) and total VFAs (tVFA, mM). Across GTE treatment, the 1^st^ *in vitro* run had higher (p < 0.001) tGP 6–24 h and pH, but lower (p < 0.001) tVFA and A: P in comparison with the 2^nd^ *in vitro* run. During the tGP measurement, it was observed that the tGP produced every 2 h experiences a constant increase until the end of fermentation in 24 h (Figure-2) [15].

**Table-2:** Means (n = 6)* in vitro* tGP (mL), degradability (%), CH_4_ (%), NH_3_ (µg/mL), and VFA (mM) of GTD after 24 h of incubation.

Measurement	Treatment				Running time		SEM and significance (p-value)		
		
	GTE-0	GTE-140	GTE-280	GTE-560	R1	R2	Treatment	Running	Treatment × Running
tGP 6 h (mL)	8.00^a^	7.83^ab^	7.16^b^	7.41^ab^	8.04^A^	7.16^B^	0.370 (p = 0.039)	0.190 (p < 0.001)	0.308 (p = 0.076)
tGP 12 h (mL)	14.7	14.2	13.9	14.3	15.33^A^	13.25^B^	0.390 (p = 0.498)	0.275 (p < 0.001)	0.551 (p = 0.063)
tGP 18 h (mL)	20.7	19.7	19.5	20.2	21.2^A^	18.9^B^	0.533 (p = 0.414)	0.377 (p < 0.001)	0.754 (p = 0.277)
tGP for 24 h (mL)	25.5	24.1	24.1	24.9	26.7^A^	22.7^B^	0.636 (p = 0.357)	0.450 (p < 0.001)	0.900 (p = 0.427)
pH	7.24	7.35	7.26	7.24	7.39^A^	7.15^B^	0.050 (p = 0.392)	0.035 (p < 0.001)	0.071 (p = 0.576)
CH_4_ (% LEL)	17.2	9.70	9.40	9.00	10.0	12.2	2.30 (p = 0.065)	1.73 (p = 0.368)	3.25 (p = 0.491)
NH_3_-N (mg/dl)	7.95	8.18	7.94	8.09	7.60	7.90	0.520 (p = 0.112)	0.368 (p = 0.664)	0.735 (p = 0.834)
DMD (%)	40.6	42.1	41.7	43.1	39.3	42.6	3.04 (p = 0.636)	2.15 (p = 0.265)	4.49 (p = 0.207)
OMD (%)	55.4	53.4	55.8	53.4	54.8	54.2	1.64 (p = 0.597)	1.11 (p = 0.697)	2.22 (p = 0.471)
Acetic Acid (mM)	9.07	9.14	7.94	8.37	6.60^B^	10.4^A^	0.405 (p = 0.069)	0.271 (p < 0.001)	0.512 (p = 0.080)
Propionic Acid (mM)	0.304	0.314	0.277	0.295	0.278^B^	0.316^A^	0.010 (p = 0.095)	0.007 (p < 0.001)	0.013 (p = 0.293)
Isobutyric Acid (mM)	1.71^a^	1.73^a^	1.55^b^	1.62^a^	1.09^B^	2.17^A^	0.061 (p = 0.027)	0.041 (p < 0.001)	0.077 (p = 0.079)
Butyric Acid (mM)	0.420	0.459	0.396	0.425	0.408^B^	0.442^A^	0.014 (p = 0.068)	0.007 (p = 0.042)	0.020 (p = 0.083)
Isovaleric acid (mM)	0.204^ab^	0.218^a^	0.176^b^	0.210^ab^	0.142^B^	0.261^A^	0.010 (p = 0.046)	0.010 (p < 0.001)	0.013 (p = 0.158)
Valeric Acid (mM)	0.711	0.673	0.314	0.669	0.315	0.869	0.252 (p = 0.716)	0.178 (p = 0.051)	0.357 (p = 0.390)
Total VFA	13.9	12.5	10.7	11.6	8.91^B^	15.3^A^	0.922 (p = 0.063)	0.619 (p < 0.001)	1.16 (p = 0.060)
A: P	29.5	28.6	28.4	28.0	24.0^B^	33.0^A^	0.925 (p = 0.510)	0.620 (p < 0.001)	1.17 (p = 0.141)

Mean values were not significantly different at p *>* 0.05 and highly significantly different at p *<* 0.001; n=Number of replicates; SEM=Standard error of mean; n/a=Not available, VFA=Volatile fatty acids, GTE=Green tea extract, DMD=Dry matter degradability, OMD=Organic matter degradability, tGP=total gas production A:P*=*Acetate-to-propionate ratio

## Discussion

The bioactive components of GTE have garnered attention for their potential health benefits, particularly when used as a dietary supplement for ruminant livestock. Caffeine is a well-known stimulant with potential implications for ruminant metabolism and performance. The most important benefits of phenolic compounds and caffeine are the inhibition of lipid peroxidation [17, 18]. EGCG, the most abundant catechin in GTE, possesses potent antioxidant properties and has been associated with various health benefits [19]. Epigallocatechin and epicatechin gallate are also noteworthy constituents of GTE. These catechins have been implicated in modulating rumen microbial populations and fermentation dynamics [20]. Similar to caffeine, theobromine may exert stimulatory effects on ruminant metabolism, whereas rutin, a flavonoid, has antioxidant properties that could enhance ruminant health and resilience against oxidative stress. Although the concentrations of compounds, such as theaflavins, are relatively low in GTE, their potential impact on ruminant health should not be overlooked.

Tannins are a class of polyphenolic compounds found in plants that are known for their astringent taste and diverse biological activities [21]. These compounds play crucial roles in plant defense mechanisms against herbivory, microbial infections, and oxidative stress [22]. However, their effects on ruminants, particularly on their digestive processes, have drawn significant attention due to their potential implications on animal nutrition and health. Ruminants can consume tannins that are beneficial and harmful. On the one hand, tannins can modulate ruminal fermentation by inhibiting the activity of fibrolytic bacteria and protozoa, thereby reducing methane emissions and improving nitrogen utilization efficiency [23]. In addition, tannins enhance protein metabolism and the absorption of essential minerals in ruminants [24].

On the other hand, excessive intake of tannins can lead to negative consequences, such as reduced feed intake, nutrient digestibility, and animal performance [25–27]. Tannins may form complexes with dietary proteins and carbohydrates, limiting their digestibility and availability for microbial fermentation in rumen [14]. This can decrease microbial protein synthesis and energy supply to host animals, ultimately affecting growth and productivity. Furthermore, certain types of tannins, particularly condensed tannins, have been associated with antinutrient effects, such as impaired amino acid absorption, enzyme inhibition, and gastrointestinal disturbances in ruminants [28]. Chronic exposure to high tannin levels may also induce oxidative stress and liver damage in ruminants through the generation of reactive oxygen species and the formation of tannin–protein complexes [29].

The present study investigated the effects of varying doses of tea extract on tGP, degradability, pH, and individual VFAs in an *in vitro* trial. The tendency to decrease gas production as the dose increases can be caused by the inhibitory effect of polyphenols on the microbial fermentation process [27]. Polyphenols affect the microbial cell walls and inhibit enzyme activity, leading to reduced microbial activity and gas production [14]. This agrees with previous research reporting that polyphenolic compounds effectively reduced enteric methane emissions [23]. Although in the research of Patra and Saxena [30] and Makkar [14], the addition of tannins to animal feed significantly impacted rumen fermentation, efforts were needed to overcome these negative effects. Tannins bind to proteins, potentially influencing microbial fermentation processes [31, 32].

Although not statistically significant, a downward trend can be observed in methane production. Methane production is closely related to the activities of methanogenic archaea, which use hydrogen and carbon dioxide during fermentation [33]. The presence of polyphenols is thought to disrupt the metabolic pathways of archaea, leading to reduced methane production. Tannins are known to bind to proteins, potentially affecting microbial fermentation processes [31, 32]. Due to their antimicrobial properties, tannins were anticipated to reduce gas production, VFA, and NH_3_ levels. However, the observed non-significant decreases in total VFA and gas productions suggest that other factors may have mitigated the inhibitory effects of tannins on microbial activity. One possible explanation could be the adaptation of rumen microbes to tannins over time, allowing them to maintain total VFA and gas productions along with NH_3_ outputs despite the presence of tannins.

Moreover, the absence of significant differences in digestibility could be attributed to the bypass protein effect. According to Hu and Murphy [32], bypass proteins in animal feed play an important role in increasing ruminant nutrient absorption. Bypass proteins are dietary proteins that escape ruminal degradation and are subsequently absorbed in the small intestine, bypassing microbial fermentation in the rumen. This mechanism ensures the availability of nutrients for absorption by the host animal, potentially increasing digestibility without significantly affecting rumen fermentation parameters. Significant differences between *in vitro* runs may be attributed to the microbial composition of the rumen, which is heavily influenced by diet, animal health status, and environmental conditions. Although the sheep were subjected to the same treatment, individual differences in metabolic responses could lead to variations in the rumen microbial composition [34]. The enzymatic activity of RF may vary among individuals. These enzymes play crucial roles in the fermentation process and in the production of gases and VFA. Variations in enzymatic activity can result in differences in *in vitro* test outcomes [35].

Interestingly, although the *in vitro* trial did not show significant improvements in rumen fermentation parameters, it is noted that *in vivo* studies have reported an increase in performance [36–38]. This inconsistency between *in vitro* and *in vivo* results suggests that complex interactions among dietary components, rumen microbes, and host physiology cannot be fully replicated *in vitro* using only rumen. The digestive process after rumen consumption is believed to also play a role in the digestion of tannins.

Further research, particularly *in vivo* studies, is warranted to elucidate the mechanisms underlying the observed effects of tea extract on rumen fermentation and animal performance. Digestibility was not significant because the fermentation time was only 24 h. This indicates that within 24 h of fermentation in the rumen, the feed given has not been fully digested. In the ruminant digestive system, the feed consumed undergoes a fermentation process by microorganisms in the rumen for several hours before being further broken down and its nutrients absorbed by the animal’s body. A short fermentation time may indicate that some feed components are not digested properly at the initial stage of fermentation. This can be caused by various factors, including feed composition, crude fiber levels, availability of digestive enzymes, and the composition of the rumen microbiota.

## Conclusion

GTE treatment tends to reduce CH_4_ production in the rumen, likely because polyphenols disrupt microbial activity. The non-significant reductions in total VFA and gas production suggest potential microbial adaptations, allowing total VFA and gas production along with NH_3_ outputs to remain stable.

## Authors’ Contributions

RSR: Conceptualization, formal analysis, and drafted and edited the manuscript. DR: Conceptualization, methodology, supervision, validation, and edited the manuscript. IH and AJ: Conceptualization, methodology, supervision, and validation. NH: Conceptualization, methodology, and reviewed and edited the manuscript. All authors have read, reviewed, and approved the final manuscript.
